# Decreased pyruvate dehydrogenase activity in Tafazzin-deficient cells is caused by dysregulation of pyruvate dehydrogenase phosphatase 1 (PDP1)

**DOI:** 10.1016/j.jbc.2024.105697

**Published:** 2024-01-30

**Authors:** Zhuqing Liang, Tyler Ralph-Epps, Michael W. Schmidtke, Vikalp Kumar, Miriam L. Greenberg

**Affiliations:** Department of Biological Sciences, Wayne State University, Detroit, Michigan, USA

**Keywords:** caridiolipin, tafazzin, Barth syndrome, metabolism, pyruvate dehydrogenase, pyruvate dehdrogenase phosphatase, calcium

## Abstract

Cardiolipin (CL), the signature lipid of the mitochondrial inner membrane, is critical for maintaining optimal mitochondrial function and bioenergetics. Disruption of CL metabolism, caused by mutations in the CL remodeling enzyme TAFAZZIN, results in the life-threatening disorder Barth syndrome (BTHS). While the clinical manifestations of BTHS, such as dilated cardiomyopathy and skeletal myopathy, point to defects in mitochondrial bioenergetics, the disorder is also characterized by broad metabolic dysregulation, including abnormal levels of metabolites associated with the tricarboxylic acid (TCA) cycle. Recent studies have identified the inhibition of pyruvate dehydrogenase (PDH), the gatekeeper enzyme for TCA cycle carbon influx, as a key deficiency in various BTHS model systems. However, the molecular mechanisms linking aberrant CL remodeling, particularly the primary, direct consequence of reduced tetralinoleoyl-CL (TLCL) levels, to PDH activity deficiency are not yet understood. In the current study, we found that remodeled TLCL promotes PDH function by directly binding to and enhancing the activity of PDH phosphatase 1 (PDP1). This is supported by our findings that TLCL uniquely activates PDH in a dose-dependent manner, TLCL binds to PDP1 *in vitro*, TLCL-mediated PDH activation is attenuated in the presence of phosphatase inhibitor, and PDP1 activity is decreased in *Tafazzin*-knockout (TAZ-KO) C2C12 myoblasts. Additionally, we observed decreased mitochondrial calcium levels in TAZ-KO cells and treating TAZ-KO cells with calcium lactate (CaLac) increases mitochondrial calcium and restores PDH activity and mitochondrial oxygen consumption rate. Based on our findings, we conclude that reduced mitochondrial calcium levels and decreased binding of PDP1 to TLCL contribute to decreased PDP1 activity in TAZ-KO cells.

Cardiolipin (CL) is a unique phospholipid with four fatty acid chains and two phosphate head groups. Following synthesis in the inner mitochondrial membrane (IMM), CL undergoes a specialized remodeling process by which its predominantly saturated fatty acid chains are progressively replaced by polyunsaturated fatty acids. The importance of the CL remodeling pathway is underscored by the life-threatening disease Barth syndrome (BTHS), in which patients bear mutations in *TAFAZZIN*, the transacylase responsible for adding polyunsaturated fatty acid chains to monolyso-CL (MLCL) (1, 2, 3). As a result, the biochemical hallmarks of BTHS include a marked decrease in unsaturated CL species, an overall decrease in CL, and a concomitant accumulation of MLCL. The functional benefit of CL remodeling is not well understood, with some studies proposing that remodeled CL does not confer a fitness advantage compared to unremodeled CL in yeast (4, 5).

In mammals, the process of CL remodeling predominantly involves the replacement of oleic acid (18:1) fatty acid chains with linoleic acid (18:2), resulting in the formation of (18:2)_4_ tetralinoeleoyl-CL (TLCL) (6, 7). TLCL is the most abundant CL species in various mammalian tissues, including the human heart, lymphoblasts, and skeletal muscle (7, 8, 9, 10, 11, 12). Reduced levels of TLCL have been linked to mitochondrial dysfunction, whereas a linoleate-rich high-fat diet has been shown to improve mitochondrial function and reduce mortality in a hypertensive heart failure rat model (13). While the functional distinction between TLCL and other CL species remains enigmatic, one possibility is that the acyl chain composition of CL may differentially influence lipid-protein interactions. Studies utilizing ^31^P-NMR spectroscopy have revealed that CL and MLCL exhibit distinct lipid-protein associations (14, 15). This disparity has also been observed in an *in vitro* protein-lipid binding assay, wherein bovine heart-derived CL, but not MLCL, was shown to interact with pyruvate dehydrogenase (PDH) (16). CL profiles also differ between organisms and show a general taxonomic trend towards increased incorporation of unsaturated fatty acid chains in CL from higher eukaryotes (17). CL homeostasis can be disturbed by disease conditions, including cancer (18), nonalcoholic fatty liver disease (19), Alzheimer’s disease (18, 20) and heart failure (21). It is poorly understood why cells maintain such a broad spectrum of CL species and what the functional difference is between specific species.

We have previously reported that PDH activity and synthesis of acetyl-CoA are decreased in CL-deficient yeast and mammalian cells (16, 22), suggesting a potential regulatory role for CL in modulating PDH activity. PDH is a crucial metabolic hub that facilitates the oxidative decarboxylation of pyruvate, resulting in the formation of acetyl-CoA, CO_2_, and NADH for utilization in the tricarboxylic acid (TCA) cycle and oxidative phosphorylation (23). The mammalian PDH complex consists of multiple copies of the three enzymatic subunits: pyruvate dehydrogenase (E1), dihydrolipoamide acetyltransferase (E2), and dihydrolipoamide dehydrogenase (E3). The activity of the PDH complex is regulated by PDH kinases (PDK) and PDH phosphatases (PDP) (24, 25, 26). Inactivation of PDH occurs when any of the three serine residues on the E1 subunit is phosphorylated by PDK (27). Conversely, enzyme activation occurs upon binding of PDP to the lipoyl domain of E2, followed by dephosphorylation of E1 (28, 29). In mammalian cells, two isoforms of PDP exist, including PDP1, primarily expressed in muscle tissue, and PDP2, which is most abundant in the liver (30). The presence of calcium facilitates binding between PDP1 and the lipoyl domain of E2, enhancing the dephosphorylation activity of PDP1 by over 10-fold (31).

Utilizing *Tafazzin*-knockout C2C12 mouse myoblasts (TAZ-KO), the current study identifies a direct and specific function of remodeled TLCL in binding to PDP1 and maintaining PDH activity. Our data suggest that impaired PDP1 activity in TAZ-KO cells is likely a dual consequence of reduced availability of TLCL to interact with PDP1 and decreased mitochondrial calcium levels. Intriguingly, treating TAZ-KO cells with calcium lactate (CaLac) restores PDH activity and rescues mitochondrial oxygen consumption rate, suggesting a potential novel therapeutic approach for treating BTHS.

## Results

### TLCL decreases PDH phosphorylation and rescues PDH activity in TAZ-KO cells

BTHS is caused by mutations in the CL remodeling enzyme TAFAZZIN. As a result of this remodeling defect, BTHS cells produce substantially less polyunsaturated CL and accumulate the remodeling intermediate MLCL. In mammalian cells, the predominant CL species in the heart and muscle is polyunsaturated TLCL, and several studies have suggested that BTHS pathology results specifically from a lack of TLCL enrichment in the heart (32, 33, 34). However, a mechanistic link between the lack of TLCL and BTHS pathology has not been demonstrated.

We have previously reported that acetyl-CoA synthesis is decreased in TAZ-KO cells (16). Consistent with this, the activity of PDH, which generates acetyl-CoA, is reduced in these cells (16). PDH activity deficiency has also been observed in a *Tafazzin*-knockdown mouse model (35). However, the mechanism whereby CL/TAFAZZIN regulates PDH is unknown. Insight into this mechanism came from our observation of a consistent increase in phosphorylated (inactive) PDH in TAZ-KO cells, which was rescued by supplementation of bovine heart CL (16). These data suggest that CL regulates PDH activity by decreasing PDH phosphorylation. However, how specific CL species (particularly TLCL) and *in vivo* remodeling of CL acyl chain composition modulate PDH activity remains unclear.

**Figure 1:**
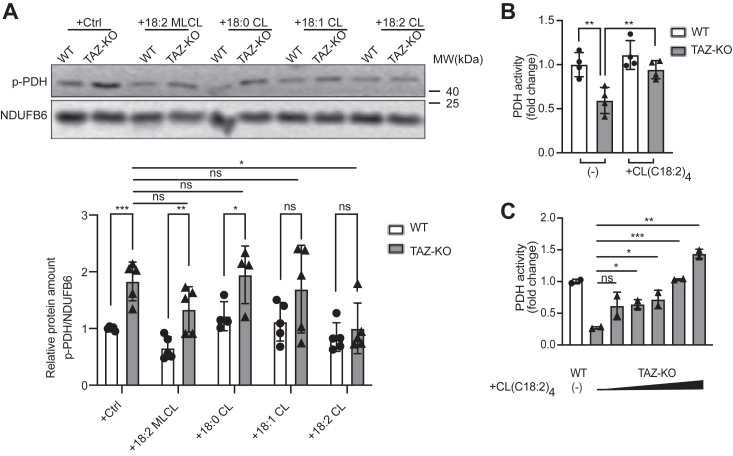
**PDH activity is rescued by supplementation of C(18:2)**_**4**_**CL.***A*, phosphorylated PDH (p-PDH) levels were assayed in mitochondria obtained from WT and TAZ-KO myoblasts. Mitochondria were incubated on ice for 2.5 h with the indicated CL species. The signal intensities of protein bands were quantified using ImageJ software (*bottom panel*). The protein band was normalized to NDUFB6 and quantified relative to the WT control-treated sample. *B* and *C*, mitochondria from WT and TAZ-KO myoblasts were treated with or without C(18:2)_4_ CL, and PDH activity was assayed as described in “Experimental procedures”. Data points represent mean ±S.D. (*error bars*) for each individual biological replicate of each group. ∗ 0.01<*p* < 0.05, ∗∗ 0.001<*p* < 0.01, ∗∗∗ <*p* < 0.001.

To determine which CL species can rescue PDH activity, we supplemented mitochondrial extracts from wild-type (WT) and TAZ-KO cells with either (18:0)_4_ CL, (18:1)_4_ CL, (18:2)_4_ CL (TLCL), or (18:2)_3_ MLCL and then assayed PDH phosphorylation and activity. Although total PDH levels were unchanged between treatment conditions (data not shown), TLCL significantly reduced the inhibitory phosphorylation of PDH (Fig. 1*A*) and restored PDH activity in TAZ-KO mitochondria in a dose-dependent manner (Fig. 1, *B* and *C*). Supplementation of saturated or (18:2)_3_ MLCL did not significantly reduce PDH phosphorylation, suggesting that remodeling of CL, and thereby enrichment of TLCL, is critical for maintaining optimal PDH activity. Despite the presence of three polyunsaturated linoleic acid acyl chains (18:2)_3_ on MLCL (identical to the four acyl chains of TLCL), it did not rescue PDH activity, indicating that the number of acyl chains *per se* is also an important determinant in the ability of CL to restore PDH function.

### TLCL-mediated rescue of PDH activity is dependent on PDP1

**Figure 2:**
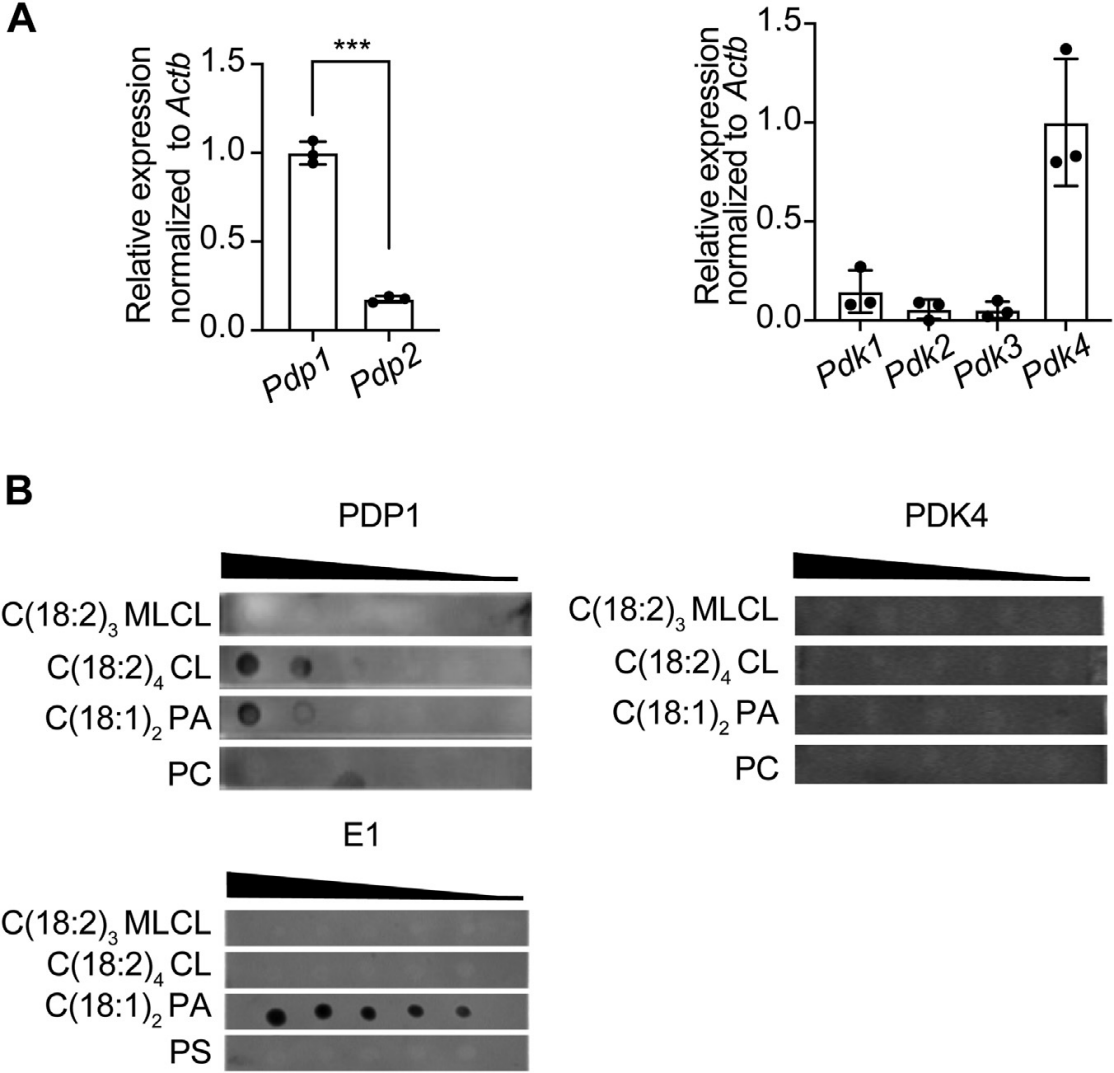
**C(18:2)**_**4**_**CL binds to PDP1 *in vitro*.***A*, PDP and PDK isoform mRNA levels were evaluated in WT myoblasts by qPCR. Expression levels were normalized to *Actb* as an internal control. Data points represent mean ±S.D. (*error bars*) for each individual biological replicate of each group. ∗∗∗ <*p* < 0.001. *B*, the indicated CL species, phosphatidic acid (PA), phosphatidylcholine (PC), and phosphatidylserine (PS), were serially diluted and spotted onto a PVDF membrane that was incubated overnight in buffer containing 25 μg of the indicated recombinant proteins PDP1. Interactions were detected by immunoblotting with an antibody against the His tag.

CL physically interacts with a myriad of different proteins in addition to the PDH complex (16, 36). Because the acyl chain structure of CL is predicted to influence its binding dynamics, we hypothesized that the unique ability of TLCL to restore PDH activity may result from its binding affinity for components of the PDH complex and its associated regulatory enzymes PDK and PDP. To this end, we performed a protein-lipid overlay assay incorporating different PDH/PDK/PDP components to test their interaction with TLCL. PDP1 and PDK4 are the predominant isoforms of PDP and PDK expressed in C2C12 myoblasts (37) (Fig. 2*A*) and were therefore the isoforms tested in this analysis. Intriguingly, only PDP1 was shown to bind with TLCL (Fig. 2*B*), suggesting that restoration of PDH activity may result from the direct interaction between TLCL and PDP1. Although PA is also bound to PDP1 (and to E1), we have previously demonstrated that PA does not restore PDH activity in TAZ-KO mitochondrial extracts (16).

**Figure 3:**
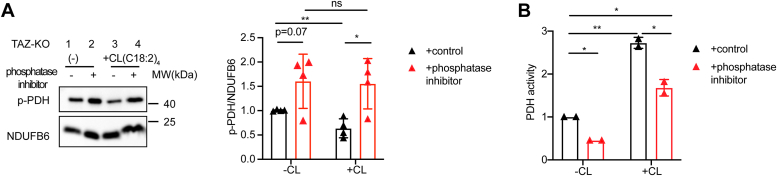
**PDP1 activity is required for TLCL-mediated rescue of PDH activity.***A*, phosphorylated PDH (p-PDH) levels and PDH activity (*B*) were evaluated in mitochondria from TAZ-KO myoblasts treated with or without phosphatase inhibitor and supplemented for 2.5 h with or without C(18:2)_4_ CL. Data points represent mean ±S.D. (*error bars*) for each individual biological replicate of each group. ∗ 0.01<*p* < 0.05, ∗∗ 0.001<*p* < 0.01.

To further investigate whether restoration of PDH activity by TLCL is dependent on PDP1, we tested the ability of TLCL to activate PDH in the presence of the serine/threonine phosphatase inhibitor PhosSTOP (38). Our results showed that PDP1 inhibition attenuated TLCL-mediated activation of PDH in TAZ-KO mitochondria (Fig. 3, *A* and *B*). As expected, TLCL supplementation alone resulted in decreased inhibitory phosphorylation of PDH (p-PDH) (Fig. 3*A*, lane 1 *vs.* 3). However, pretreating cells with phosphatase inhibitor blocked the ability of TLCL to reduce p-PDH levels (Fig. 3*A*, lane 3 *vs.* 4). The observations regarding phosphorylation of PDH were mirrored in PDH activity, where pretreatment with phosphatase inhibitor substantially reduced PDH activity in TLCL-supplemented cells (Fig. 3*B*). Taken together, these findings suggest that remodeled TLCL enhances PDH activity by binding to PDP1, thereby facilitating the dephosphorylation of PDH-E1.

### Binding of PDP1 to PDH is reduced in TAZ-KO cells

**Figure 4:**
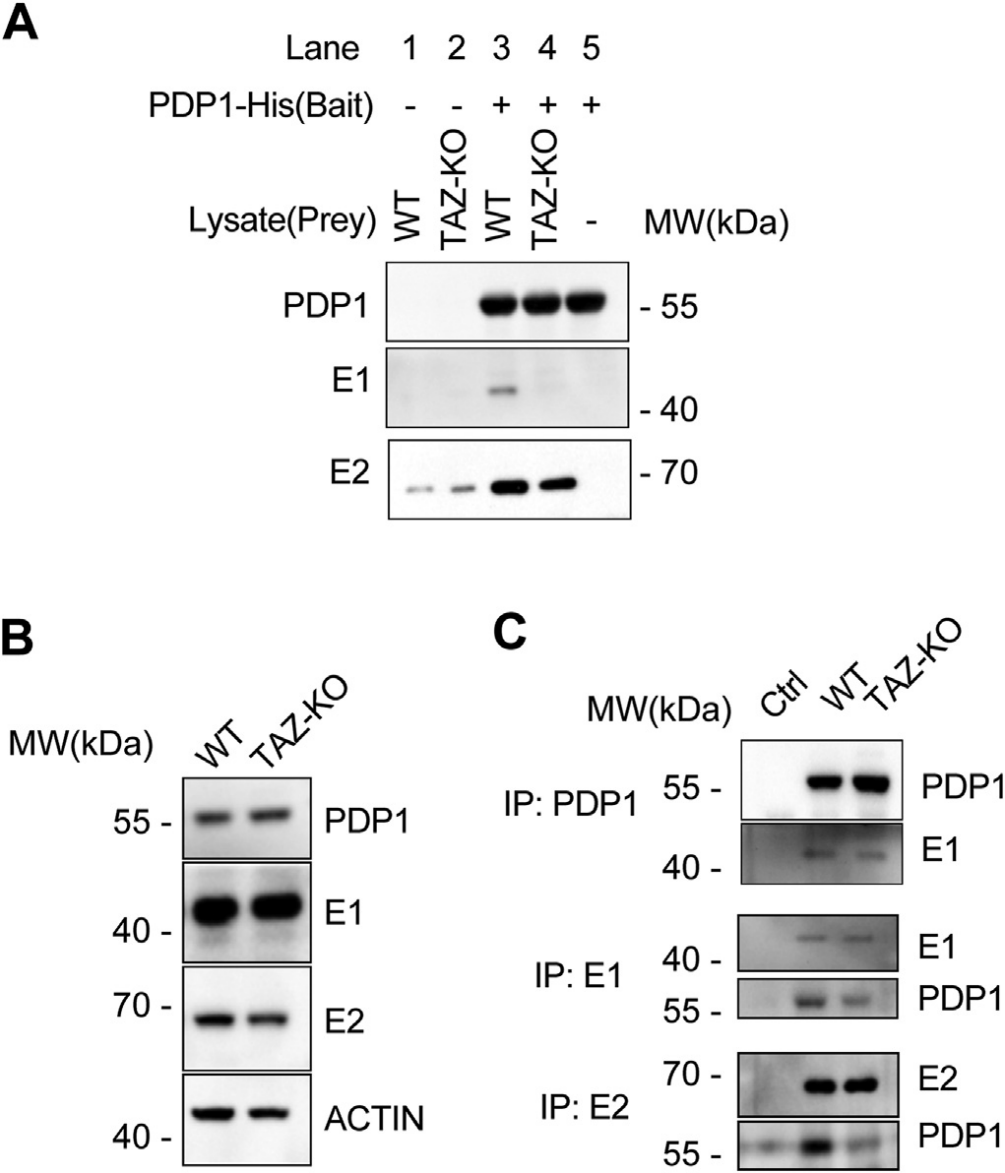
**The binding of PDP1 to PDH is reduced in TAZ-KO cells.***A*, WB analysis of the bait-prey pull-down assay. *B*, cell lysates from WT and TAZ-KO cells were subjected to WB analysis using the indicated antibodies. *C*, co-immunoprecipitation assay for PDP1, PDH-E1, and PDH-E2 protein interaction. The indicated myoblast lysates were immunoprecipitated using the antibodies specified on the left side of the figure, and eluted protein was subjected to WB analysis by probing for the specific proteins listed on the right side. The negative control lane represents WT lysate passed through empty beads lacking primary antibodies.

Next, we examined the reasons for decreased PDH phosphorylation in TAZ-KO cells upon treatment with TLCL. The previous experiment suggests that restoration of PDH activity by TLCL is dependent on PDP1 (Fig. 3). Therefore, we hypothesized that TLCL enhances the activity of PDP1. PDP1 activates PDH by first binding to the lipoyl domain of PDH-E2 and then subsequently binding to the PDH-E1 subunit to dephosphorylate the key regulatory serine residues (28, 29, 39, 40). To test the hypothesis that TLCL facilitates binding of PDP1 to PDH-E1, we employed two approaches. First, we used recombinant PDP1 protein as bait and performed a pull-down assay using WT and TAZ-KO cell lysates. Our results revealed decreased interaction between PDP1 and both the E1 and E2 subunits of PDH in TLCL-deficient TAZ-KO cells (Fig. 4*A*), despite no significant difference in steady-state levels of PDP1 protein (Fig. 4*B*). As a complementary approach, we implemented co-immunoprecipitation (Co-IP) and consistently observed a decrease in the interaction between endogenous PDP1 and both E1 and E2 in TAZ-KO lysates (Fig. 4*C*). These findings indicate that the binding of PDP1 to PDH subunits E1 and E2 is decreased in TAZ-KO cells, supporting a mechanism whereby diminished TLCL levels result in a lower affinity of PDP1 for its PDH substrate.

### PDP1 activity is reduced in TAZ-KO cells

**Figure 5:**
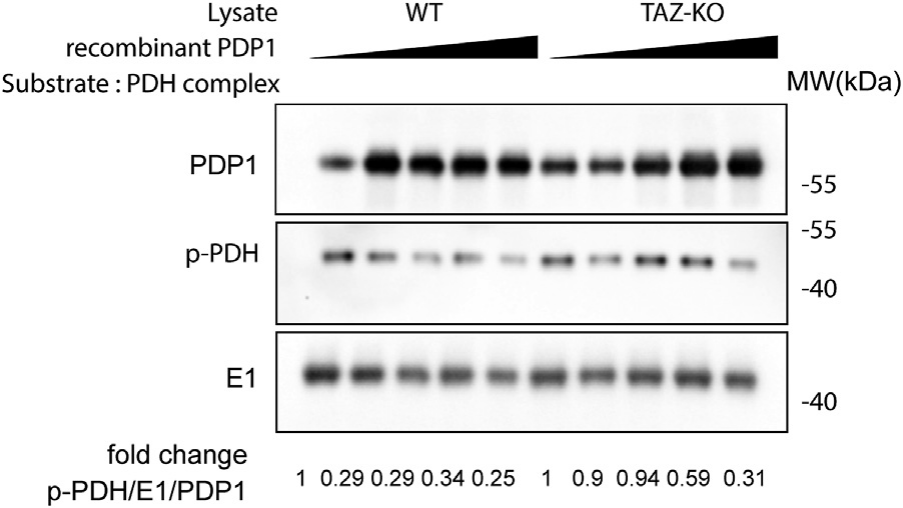
**PDP1 activity is decreased in TAZ-KO cells.** Increasing amounts of recombinant PDP1 protein were incubated with WT or TAZ-KO lysates. PDP1 eluted from this mixture was subsequently incubated with PDH complex for 1 h. The dephosphorylation activity of PDP1 was evaluated by assaying phosphorylated PDH (p-PDH) using WB analysis. p-PDH levels were quantified by dividing the amount of p-PDH signal by total PDH-E1 in each sample, and values were normalized relative to the first lane in each group (containing the lowest concentration of eluted PDP1).

Based on the previously reported findings that TLCL levels and PDH activity are substantially reduced in TAZ-KO cells (16, 37) and our data demonstrating that TLCL enhances PDH activity in a PDP1-dependent manner (Fig. 3), we predicted that PDP1 activity is decreased in TAZ-KO cells. To directly test PDP1 activity, we employed an *in vitro* PDP1 activity assay modified from Shan *et al*., (41). In this assay, we incubated increasing amounts of recombinant PDP1 protein with commercial PDH complex (Sigma, P7032) following incubation with WT or TAZ-KO lysates. To validate the system, we first confirmed that the addition of recombinant PDP1 to PDH effectively resulted in reduced levels of phosphorylated PDH relative to PDP1-free controls. We then tested our hypothesis by assaying PDH phosphorylation following the addition of recombinant PDP1 protein that had been previously incubated with either WT or TAZ-KO lysates. We observed decreased PDP1-mediated PDH dephosphorylation following incubation of PDP1 with TAZ-KO cell lysate. Specifically, incubation of PDP1 with WT lysate resulted in a ∼70% reduction in PDH phosphorylation vs. only a ∼10% reduction following TAZ-KO lysate incubation (Fig. 5). To achieve a similar degree of PDH dephosphorylation, 30% more PDP1 protein was required in the reaction mixture when it had been previously incubated with lysate from TAZ-KO cells vs. WT cells. This suggests that PDP1 is modified in the TAZ-KO intracellular environment, resulting in diminished *in vitro* enzyme activity. Notably, the PDH phosphorylation measured in this assay reflected only that of the exogenous PDH complex, as levels of endogenous PDH-E1 pulled down from cell lysates during the PDP1 incubation were negligible (data not shown).

Taken together, these findings suggest that PDP1 activity is reduced in TAZ-KO cells, as evidenced by decreased binding affinity between PDP1 and its PDH substrate, decreased *in vitro* dephosphorylation activity, and abrogated rescue of PDH activity and phosphorylation in TLCL-supplemented mitochondria pretreated with phosphatase inhibitor.

### Mitochondrial calcium levels are decreased in TAZ-KO cells

Calcium enhances the activation of PDP1 by increasing its binding affinity for PDH-E2 (29, 38, 42). Therefore, impaired binding of PDP1 to PDH-E1/E2 suggests a potential secondary mechanism of PDP1 inhibition in TAZ-KO cells resulting from decreased mitochondrial calcium levels. In support of this, a recent study demonstrated that the expression of proteins associated with the mitochondrial calcium uniporter (10.13039/100009915MCU) is substantially reduced in BTHS patient-derived tissues and that MCU-mediated calcium uptake is impaired in TAZ-KO C2C12 myoblasts (43).

**Figure 6:**
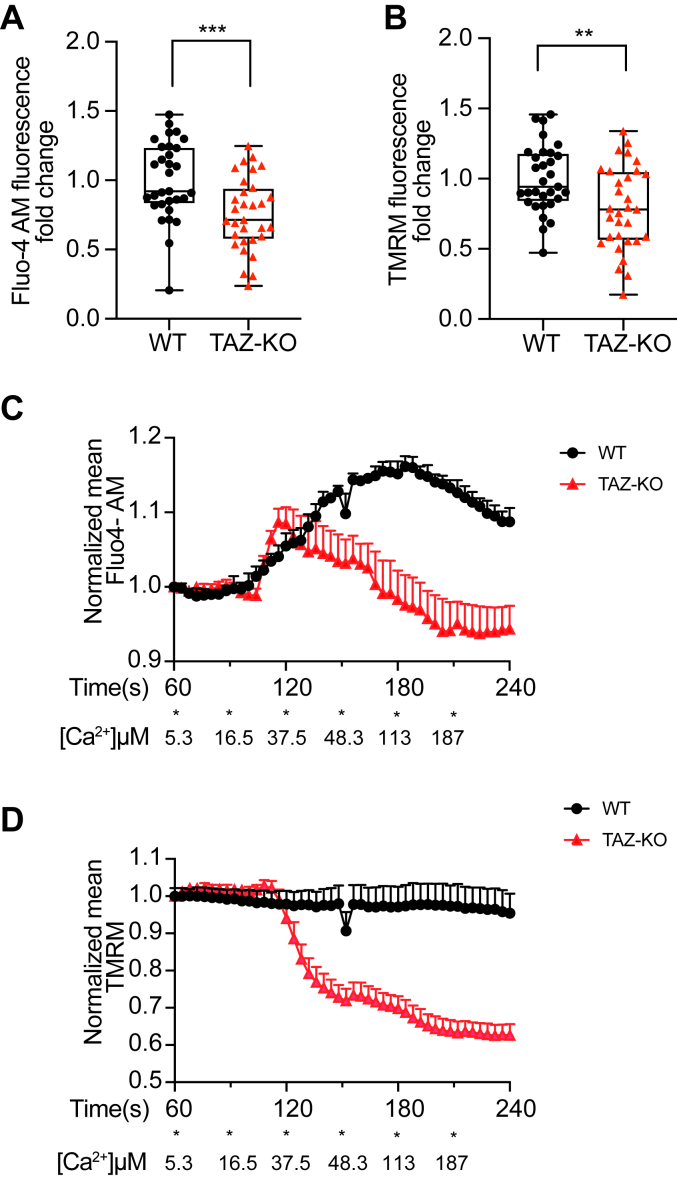
**TAZ-KO mitochondria exhibit decreased calcium levels.** WT and TAZ-KO cells were preloaded with Fluo-4 AM and TMRM before permeabilization with 50 μg/ml (w/v) digitonin and 1 μM thapsigargin. Steady-state quantification of Fluo-4 AM (*A*) and TMRM (*B*) signals in WT and TAZ-KO mitochondria was performed using Image J software. n = 31. Subsequently, WT and TAZ-KO mitochondria were injected with 5 mM CaCl_2_ (100 μl) into the 250 μl imaging solution at the indicated time points (indicated as each ∗, with the resulting effective [Ca^2+^] listed below). Fluo-4 AM (*C*) and TMRM (*D*) density were quantified over time. Data points represent mean ±S.D. (*error bars*) for each individual biological replicate of each group. ∗∗ 0.001<*p* < 0.01, ∗∗∗ <*p* < 0.001.

To further investigate mitochondrial calcium uptake and concentration, we titrated cells with calcium chloride to increase intracellular calcium levels while simultaneously tracking Fluo-4 AM (calcium indicator) and TMRM (mitochondrial membrane potential (MMP) probe) fluorescence by confocal microscopy (44, 45). To precisely measure mitochondrial calcium, cells were permeabilized to clear cytosolic calcium and imaged in an intracellular buffer containing 1 μM thapsigargin, which eliminates ER calcium signals by inhibiting the predominant ER Ca^2+^ ATPase (46). Steady-state analysis revealed decreased mitochondrial calcium signals in TAZ-KO mitochondria compared to WT (Fig. 6*A*). TMRM analysis demonstrated that the MMP is also reduced in TAZ-KO cells, which is consistent with our previous observation (Fig. 6*B*) (47). Using this methodology, calcium uptake can be visualized by an increase in mitochondrial matrix Fluo-4 AM signal. TAZ-KO cells showed no significant difference in the rate of mitochondrial calcium uptake relative to WT through 120 s of imaging (Fig. 6*C*). Interestingly, after the gradual addition of calcium, TAZ-KO cells showed a drop in the TMRM signal at approximately 120 s of imaging (Fig. 6*D*). The concomitant drop in TAZ-KO mitochondrial Fluo-4 AM signal at this timepoint reflects the rapid loss of mitochondrial calcium following the loss of membrane potential. These data indicate that the decreased levels of mitochondrial matrix calcium in TAZ-KO cells are not due to decreased mitochondrial calcium uptake but a reduced capacity to maintain calcium levels as high as WT cells. In summary, these data suggest that decreased PDP1 activity and binding affinity for PDH-E2 are due, at least in part, to reduced mitochondrial calcium levels in TAZ-KO cells.

### CaLac treatment rescues PDH activity and OCR in TAZ-KO cells

In light of our findings that TAZ-KO cells have reduced mitochondrial calcium levels, we sought to test if restoring mitochondrial calcium would rescue PDH activity. To test this, we treated cells with calcium lactate (CaLac), a compound that passively diffuses across cell membranes and increases free calcium levels, thereby circumventing deficiencies in intracellular calcium transport (48).

**Figure 7:**
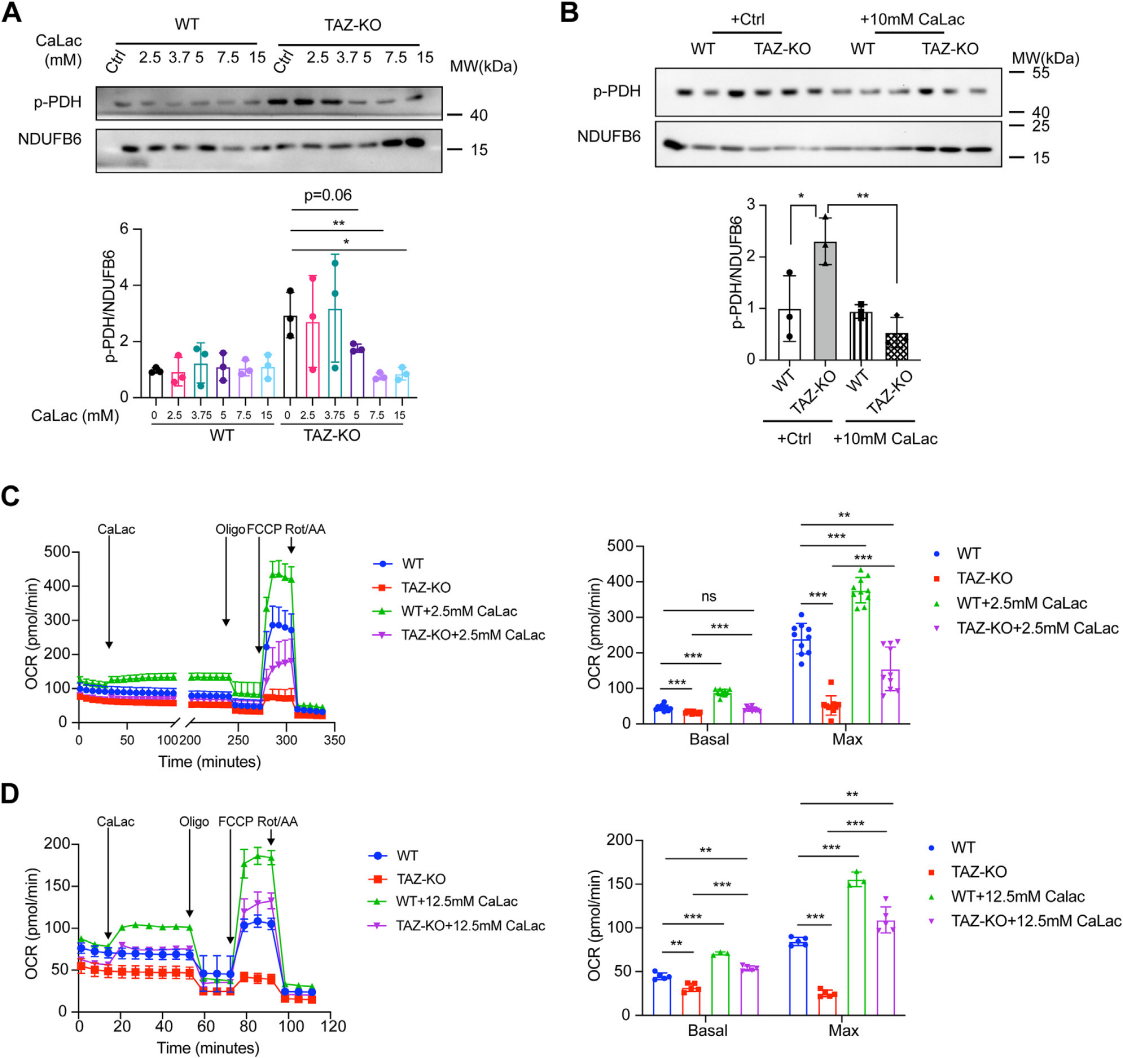
**CaLac treatment restores PDH activity and rescues OCR in TAZ-KO cells.** Phosphorylated PDH (p-PDH) levels were measured by WB following treatment with calcium lactate (CaLac) at the indicated concentrations (*A*) and with 10 mM CaLac (*B*). Oxygen consumption rates (OCR) of myoblasts subjected to 2.5 mM CaLac (*C*) and 12.5 mM CaLac (*D*) treatments. n = 5. Data points represent mean ±S.D. (*error bars*) for each individual biological replicate of each group. ∗ 0.01<*p* < 0.05, ∗∗ 0.001<*p* < 0.01, ∗∗∗ <*p* < 0.001.

Treatment of TAZ-KO cells with CaLac resulted in a reduction in PDH phosphorylation (Fig. 7, *A* and *B*). Moreover, the oxygen consumption rate (OCR) was increased in TAZ-KO cells treated with CaLac. Even at a concentration of 2.5 mM CaLac, basal OCR was restored to WT levels (Fig. 7*C*), and a higher concentration of CaLac (12.5 mM) further enhanced the OCR beyond WT levels (Fig. 7*D*). These findings indicate that increasing mitochondrial calcium levels improves PDH activity and mitochondrial respiratory function in TAZ-KO cells.

## Discussion

In this study, we highlight the significance of CL remodeling to maintain the function of PDH, the gatekeeping enzyme for the TCA cycle. PDP1 activity, which activates PDH, is reduced in TAZ-KO mitochondria. We hypothesize that at least two factors contribute to PDP1 inhibition in TAZ-KO cells—ecreased TLCL, which binds PDP1 and likely enhances its interaction with PDH, and decreased mitochondrial calcium, which has been shown to inhibit PDP1 binding and activation (29, 38, 42).

Many studies aimed at understanding TAFAZZIN deficiency in the context of BTHS have focused on indirect downstream phenotypes without identifying the primary/direct consequence of impaired CL remodeling. The *ex vivo* experiments conducted in our study identified a *direct* and *specific* role for remodeled TLCL in binding to PDP1, thereby facilitating the activation of PDH through dephosphorylation. This model is supported by our finding that when phosphatase activity is inhibited, TLCL is unable to rescue PDH activity, suggesting dependency on PDP1 activity. Although PDP1 binding and activity are enhanced by calcium (42), the ability of TLCL to rescue PDH in isolated mitochondria using a calcium-free buffer suggests that this mechanism operates independently of modulating mitochondrial calcium levels. Instead, TLCL may serve as a scaffolding molecule to promote the interaction between PDP1 and PDH, thereby facilitating PDH activation. This is supported by the observed dose-dependency of TLCL rescue, wherein increasing levels of TLCL would effectively create additional sites for PDP1 to interact with PDH.

Interestingly, we did not observe decreased calcium uptake associated with *Tafazzin* deficiency as Ghosh *et al.* reported. This discrepancy may be the result of differences in how calcium treatments were administered to cells (gradual addition of calcium in the current study vs. an acute, highly concentrated single injection of calcium by Ghosh *et al.*). However, both studies provide evidence of decreased mitochondrial matrix calcium levels in TAZ-KO cells relative to WT (43). Given the importance of calcium homeostasis, the exact nature of how decreased mitochondrial calcium affects TAZ-KO cells remains to be determined. However, it can be excluded that calcium activates PDP1 activity by facilitating the binding of PDP1 to PDH-E1, as we did not observe a difference in the binding affinity between PDP1 and PDH-E1 (as measured by immunoprecipitation) when TAZ-KO cells were treated with either exogenous calcium or the calcium chelator EGTA (data not shown). Therefore, it is likely that decreased mitochondrial calcium acts in conjunction with the reduction in TLCL to reduce the dephosphorylation of PDH-E1 by PDP1.

Treating TAZ-KO cells with CaLac rescued both PDH phosphorylation and OCR in a dose-dependent manner, suggesting that decreased calcium levels play a role in TAZ-KO-associated PDH deficiency and mitochondrial dysfunction. Interestingly, Ghosh *et al.* did not observe enhanced PDH dephosphorylation or activity following treatment with isoprenaline hydrochloride, a *β*-adrenergic agonist known to indirectly increase mitochondrial calcium uptake (43). In light of the ability of CaLac to diffuse passively across membranes (a characteristic not shared by isoprenaline hydrochloride), these two sets of findings collectively suggest that TAZ-KO cells possess a defect in maintaining mitochondrial calcium homeostasis. However, CaLac treatment has also been shown to enhance the activity of lactate dehydrogenase B (LDHB), which converts lactate into pyruvate—the primary substrate of PDH (48). Therefore, we cannot rule out the possibility that CaLac treatment enhances PDH activity in part by increasing substrate availability. However, we view this possibility as less likely, due to previous reports of both lactate and pyruvate accumulation in TAZ-KO cells (16, 37). This is also supported by our finding that cells treated with GSK2837808A, a specific lactate dehydrogenase A inhibitor that favors LDHB-mediated conversion of lactate to pyruvate, failed to activate PDH activity and rescue OCR in TAZ-KO cells (data not shown). Interestingly, treating TAZ-KO cells with 12.5 mM CaLac restored OCR to levels exceeding what was observed in untreated WT cells. Although CaLac likely enhances the activity of calcium-sensitive PDP1, the observation that phospho-PDH levels are similar between these groups suggests that CaLac may act through an additional mechanism to enhance mitochondrial function. Indeed, several enzymes in the TCA cycle and ETC are also calcium-sensitive, including isocitrate dehydrogenase, oxoglutarate dehydrogenase, cytochrome c oxidase, and ATP synthase (49). Therefore, CaLac treatment may represent an especially robust means for mitigating mitochondrial dysfunction.

Overall, this study addresses a gap in our understanding of BTHS by identifying a novel and direct role for remodeled TLCL in binding to PDP1 to promote activation of PDH. While an exact understanding of the *in vivo* binding dynamics between TLCL, PDP1, and PDH-E1/E2 will require additional studies, the current work suggests that reduced mitochondrial calcium levels likely contribute to diminished PDP1 and PDH activity in the context of *Tafazzin* deficiency.

## Experimental procedures

### Cell lines and growth conditions

The TAZ-KO C2C12 mouse myoblast model was generated previously from WT C2C12 cells using CRISPR/Cas9 targeted against exon three of the *Tafazzin* gene (37). Off-target analysis was performed based on the top 10 predicted off-target sites as previously described (16). Cells were grown in Dulbecco’s modified Eagle’s medium (DMEM; Gibco) containing 4.5 g/L glucose, supplemented with 10% fetal bovine serum (Hyclone), 2 mM glutamine (Gibco), penicillin (100 units/ml), and streptomycin (100 μg/ml). All cell lines were incubated in 5% CO_2_ at 37 °C.

### Mitochondrial extraction

Cells (1X10^7^) were washed once with PBS and homogenized with a glass homogenizer in mitochondrial isolation buffer (280 mM sucrose, 0.25 mM EDTA, 0.02 M Tris (pH 7.4)). Cell debris was removed by centrifugation at 720*g* for 5 min. Mitochondria were subsequently collected by centrifugation at 14,812*g* for 10 min. Protein concentration was determined by Bradford assay (Bio-Rad).

### CL supplementation and PDH activity

Mitochondria isolated from 1X10^7^ myoblasts were suspended in a 1:1 ratio of 50 mM NaCl and 50 mM imidazole (pH 7.4). Individual CL species were prepared fresh in 50% ethanol prior to each experiment. For every 100 μg of mitochondria, 1.5 μl of 10% digitonin (protein/digitonin ratio 100:150) was added, followed by the addition of 1 μl of CL (20 mg/ml) or 4 μl of CL (5 mg/ml) or 50% ethanol (control). The interaction was kept on ice for 2.5 h, and then 2 μl of 10% digitonin was added to each group to reach a final protein/digitonin ratio of 100:350. For PDH activity, 10 to 30 μg of lysate was used with a PDH activity assay kit (MAK183, Sigma) at 37 °C. The mitochondrial lysate was extracted with the addition of a phosphatase inhibitor in the corresponding assay (PhosSTOP, Roche).

### RNA extraction and real-time qPCR analysis

cDNA was synthesized from total RNA using SuperScript (Invitrogen). Real-time qPCR was carried out with the QuantSudio3 real-time qPCR machine (Applied Biosystems) using PowerUp SYBR Green reagents (Thermo Fisher). Relative mRNA transcript levels were quantified with the 2–ΔΔCT method (where CT is the threshold cycle), using *Actb* as an internal control. Primers used in this study are listed in Table 1.

**Table 1:** qPCR Primers used in this paper

Gene	Forward	Reverse
*Actb*	GGTCGTACCACAGGCATTGTGATG	GGAGAGCATAGGCCTCGTAGATGG
*Pdp1*	ATGCCAGCACCAACTCAACT	GGGTGTGTACCTCAGACGATT
*Pdp2*	ACTGTGTCCTACTGGATCTTCAA	CAGGTTCCTACTCGTGGCA
*Pdk1*	GGACTTCGGGTCAGTGAATGC	TCCTGAGAAGATTGTCGGGGA
*Pdk2*	AGGGGCACCCAAGTACATC	TGCCGGAGGAAAGTGAATGAC
*Pdk3*	TCCTGGACTTCGGAAGGGATA	GAAGGGCGGTTCAACAAGTTA
*Pdk4*	CGCTTAGTGAACACTCCTTCG	CTTCTGGGCTCTTCTCATGG

### Construction of bacterial expression vector for PDP1 cDNA

Methods were adapted and modified from the protocol by Huang *et al*. (30). Briefly, the coding region of PDP1 cDNA corresponding to the mature polypeptide was constructed by PCR using cDNA as a template and native Q5 Hot Start High Fidelity Polymerase (NEB). The resulting PCR product contained *Nco*I and *Xho*I restriction sites flanking the coding region. The forward primer (5′-AAA CCA TGGCTT CTA CAC CGC AGA AAT TTT AC-3′), corresponding to bases 215 to 237 of PDP1 cDNA, included the *Nco*I restriction site. The reverse primer (5′-TTT CTC GAG CTG TTC CTG GTT TTG GTA TGC-3′), corresponding to bases 1594 to 1664, contained the *Xho*I restriction site. The primers were designed based on the transcript sequenced archived on the Ensembl website (MGP_C3HHeJ_T0064368.1). The resulting cDNA was digested with *Nco*I and *Xho*I endonucleases and subcloned between the *Nco*I and *Xho*I sites of the pET-28a expression vector to produce a carboxyl-terminal fusion with His_6_ tag encoded by the vector (plasmid PDP1). The plasmids were sent for sequencing to confirm the correct orientation of PDP1 (Genewiz).

### Expression and purification of PDP1 protein

PDP1 and pGro7 (Takara) were co-transformed into BL21 (DE3) competent cells. The resulting transformants were then selected on LB agar plates containing kanamycin (100 μg/ml) and chloramphenicol (34 μg/ml). The cells were pre-cultured the day before and diluted by 1:50 with 100 ml of fresh cell medium containing 0.1% L-arabinose. The cells were then incubated at 37 °C with constant shaking at 230 rpm the next day until the OD_600_ of the culture reached 0.5 to 0.7. The cells were induced with 0.4 mM β-D-thiogalactopyranoside and incubated for another 2 h at 37 °C. The cells were then harvested and resuspended in lysis buffer containing 50 mM HEPES, 150 mM NaCl, 20 mM imidazole supplemented with 0.4 mM phenylmethylsulfonyl fluoride (PMSF), 0.5% TritonX100, and 10 mM β-mercaptoethanol. The cells were sonicated on ice to avoid foaming or excessive heat. The extraction was clarified by removing inclusion bodies at 600*g* for 30 min at 4 °C. The supernatant was purified and concentrated using nickel resin (HisPur Ni-NTA, Thermo Scientific) and centrifugal filters (Amicon Ultra, Millipore).

### PDP1 pull down and protein immunoprecipitation

Purified PDP1-His_6_ was immobilized on HisPur resin columns (Pierce Pull-down polyHis protein: protein interaction Kit, Thermo Scientific). WT and TAZ-KO myoblasts (2 × 10^6^) were lysed in a buffer containing 25 mM Tris, 75 mM NaCl, and 0.1% Tween 20 (pH 7.4) and washed once with PBS. After lysing on ice for ∼30 min, the cells were briefly sonicated and centrifuged to obtain a clarified supernatant that was then incubated overnight at 4 °C in a column with immobilized PDP1-His. The next day, the column was washed 5 times before elution. The lysates were then used for immunoprecipitation by using specific antibodies. PDH-E1, 1:500 (sc-377092, SantaCruz), PDH-E2, 1:2000 (13426-1-AP, Proteintech), ACTIN, 1:1000 (sc47778, Santa Cruz); PDP1, 1:1000 (65,575, Cell Signaling).

### PDP1 dephosphorylation assay

Eluted PDP1 from the PDP1 pull-down assay was further incubated with purified PDH complex in PDP1 buffer containing 10 mM HEPES, pH 7.5, 100 μM EDTA, 0.5% BSA, and 1 mM DTT at room temperature (41). The dephosphorylation of Ser^293^ of PDH was tested using p-PDH (Ser293), 1:1000 (AP1062; Millipore).

### Protein-lipid overlay assay

Protein lipid overlay assays were conducted following the method outlined by Dowler *et al.* with modifications (50). Different species of CL (Avanti 850,081, 710,334, 710,335, 791,108), phosphatidylcholine (PC, Avanti 840,051), phosphatidylserine (PS, Sigma P7769), and phosphatidic acid (Avanti 840,875) were dissolved in a solution of methanol and chloroform (1:1) to create 1 mM stocks. Subsequently, the lipids were diluted to various concentrations (500, 250, 125, 62.5, and 31.25 μM) in a mixture of methanol:chloroform:water (2:1:0.8). Each dilution of the lipid (1 μl) was then applied to a polyvinylidene fluoride (PVDF) membrane. Following a 1 h drying period at room temperature, the membranes were subjected to a blocking buffer (50 mm Tris-HCl, pH 7.5, 150 mM NaCl_2_, 0.1% Tween 20, and 3% BSA) for 1 h at room temperature with gentle rocking. The membranes were incubated overnight at 4 °C with gentle rocking in 10 ml of blocking buffer containing 25 μg of indicated protein PDP1, PDK4 (Sino Bio), or E1 (Cloud-Clone Corp). Protein binding to the membrane lipids was detected through immunoblotting using primary anti-His tag antibody 1:1000 (MA1-21315, Invitrogen) and a secondary antibody conjugated to HRP (1:10,000, Invitrogen).

### Mitochondrial Ca^2+^ uptake measurements

Myoblasts were cultured in Attofluor cell chambers for microscopy experiments (Thermo Fisher). The method was adapted and optimized in C2C12 myoblasts from McKenzie *et al*. (44). In brief, myoblasts were incubated for 45 min in 250 μl staining solution: 156 mM NaCl, 3 mM KCl, 2 mM MgSO_4_, 1.25 mM KH_2_PO4, 10 mM D-glucose, 2 mM CaCl_2,_ and 10 mM HEPES pH 7.35, 5 μg/ml (w/v) Fluo-4 AM, 0.005% TritonX100. After cells were washed with Ca^2+^-free Hanks' Balanced Salt Solution (HBSS), they were incubated with 300 μl intracellular medium containing 6 mM NaCl, 130 mM KCl, 7.8 mM MgCl_2_, 1 mM KH_2_PO4, 0.4 mM CaCl_2_, 2 mM EGTA, 10 mM HEDTA, 2 mM malate, 2 mM glutamate, 2 mM ADP, 20 mM HEPES (pH 7.1), 50 μg/ml (w/v) digitonin and 1 μM thapsigargin. The imaging process involved capturing images every 4 s using a 60X oil immersion objective fitted to a TCS SP8 confocal fluorescence microscope (Leica). The first 60 s were regarded as the baseline, followed by the addition of 100 μl of 5 mM CaCl_2_ at each indicated time point. Images were analyzed using ImageJ, where multiple regions of interest (ROI) were selected, and the fluorescence intensity was computed over different time intervals. To calculate ΔF (fluorescence change), the median intensity values were divided by the average of the first 60 s of recording per single ROI. The final free Ca^2+^ ion concentration [Ca^2+^] in the IM imaging solution was calculated using Maxchelator WEBMAXC EXTENDED (http://www.stanford.edu/%7Ecpatton/maxc.html).

### Western blot analysis

Protein was extracted in RIPA buffer with proteinase and phosphatase inhibitor cocktail (ChemCruz). 20 μg of denatured total protein for each sample was loaded into an SDS-PAGE gel and separated by electrophoresis. The gel was transferred onto PVDF membranes using a wet transfer method. After transfer, the membranes were probed with the following primary antibodies against NDUFB6, 1:10,000 (ab110244, Abcam); p-PDH (Ser293), 1:1000 (AP1062, Millipore); IP3R 1:500 (sc-271197, Santa Cruz), GRP75 1:500 (sc-133137, Santa Cruz), and VDAC1 1:500 (sc-390996, Santa Cruz). Total protein was quantified from each sample using a No-Stain labeling reagent (Invitrogen) and subsequently used for total protein normalization (TPN). Anti-mouse or anti-rabbit secondary antibodies (1:10,000, Invitrogen) were incubated for 1 h at room temperature, and membranes were developed using SuperSignal West Pico PLUS or West Atto ECL (Thermo Scientific).

### OCR measurements in intact mammalian cells

WT and TAZ-KO C2C12 myoblasts were plated in XF96-well cell culture microplates (Agilent Technologies) at 30,000 cells/well in 80 μl of DMEM complete growth medium and incubated at 37 °C, 5% CO_2_ for ∼4 h. Before OCR measurement, 200 μl of pre-warmed growth medium was added to each well, and cells were incubated at 37 °C for 30 min. OCR measurements were carried out in intact cells using a Seahorse XFe96 Extracellular Flux Analyzer (Agilent Technologies). Calcium lactate (CaLac, Sigma) was injected through port D at a final concentration of 5 μM and 2.5/12.5 μM individually. For the mitochondrial stress test, oligomycin, carbonyl cyanide p-trifluoro-methoxyphenyl hydrazone (FCCP), and antimycin A were sequentially injected through ports A, B and C to achieve final concentrations of 2 μM, 1 μM, and 0.5 μM, respectively.

### Statistical analysis

All values are presented as mean ± SD. Statistical analyses were performed in GraphPad Prism software using a two-tailed unpaired Student’s *t* test on data obtained from at least three independent experiments performed with different biological replicates.

## Data availability

All data are contained within the article.

## Conflict of interest

The authors declare that they have no conflicts of interest with the contents of this article.

## References

[1] Barth P.G., Wanders R.J., Vreken P., Janssen E.A., Lam J., Baas F. (1999). X-linked cardioskeletal myopathy and neutropenia (Barth syndrome) (MIM 302060). J. Inherit. Metab. Dis..

[2] Xu Y., Kelley R.I., Blanck T.J.J., Schlame M. (2003). Remodeling of cardiolipin by phospholipid transacylation. J. Biol. Chem..

[3] Schlame M., Ren M. (2006). Barth syndrome, a human disorder of cardiolipin metabolism. FEBS Lett..

[4] Ye C.Q., Lou W.J., Li Y.R., Chatzispyrou I.A., Huttemann M., Lee I. (2014). Deletion of the cardiolipin-specific phospholipase Cld1 rescues growth and life span defects in the tafazzin mutant implications for barth syndrome. J. Biol. Chem..

[5] Baile M.G., Sathappa M., Lu Y.W., Pryce E., Whited K., McCaffery J.M. (2014). Unremodeled and remodeled cardiolipin are functionally indistinguishable in yeast. J. Biol. Chem..

[6] Xu Y., Malhotra A., Ren M., Schlame M. (2006). The enzymatic function of tafazzin. J. Biol. Chem..

[7] Oemer G., Koch J., Wohlfarter Y., Alam M.T., Lackner K., Sailer S. (2020). Phospholipid acyl chain diversity controls the tissue-specific assembly of mitochondrial cardiolipins. Cell Rep..

[8] Hoch F.L. (1992). Cardiolipins and biomembrane function. Biochim. Biophys. Acta.

[9] McGee C.D., Lieberman P., Greenwood C.E. (1996). Dietary fatty acid composition induces comparable changes in cardiolipin fatty acid profile of heart and brain mitochondria. Lipids.

[10] Stefanyk L.E., Coverdale N., Roy B.D., Peters S.J., LeBlanc P.J. (2010). Skeletal muscle type comparison of subsarcolemmal mitochondrial membrane phospholipid fatty acid composition in rat. J. Membr. Biol..

[11] Acehan D., Vaz F., Houtkooper R.H., James J., Moore V., Tokunaga C. (2011). Cardiac and skeletal muscle defects in a mouse model of human Barth syndrome. J. Biol. Chem..

[12] Mejia E.M., Zegallai H., Bouchard E.D., Banerji V., Ravandi A., Hatch G.M. (2018). Expression of human monolysocardiolipin acyltransferase-1 improves mitochondrial function in Barth syndrome lymphoblasts. J. Biol. Chem..

[13] Chicco A.J., Sparagna G.C., McCune S.A., Johnson C.A., Murphy R.C., Bolden D.A. (2008). Linoleate-rich high-fat diet decreases mortality in hypertensive heart failure rats compared with lard and low-fat diets. Hypertension.

[14] Beyer K., Klingenberg M. (1985). ADP/ATP carrier protein from beef heart mitochondria has high amounts of tightly bound cardiolipin, as revealed by 31P nuclear magnetic resonance. Biochemistry.

[15] Eble K.S., Coleman W.B., Hantgan R.R., Cunningham C.C. (1990). Tightly associated cardiolipin in the bovine heart mitochondrial ATP synthase as analyzed by 31P nuclear magnetic resonance spectroscopy. J. Biol. Chem..

[16] Li Y., Lou W., Raja V., Denis S., Yu W., Schmidtke M.W. (2019). Cardiolipin-induced activation of pyruvate dehydrogenase links mitochondrial lipid biosynthesis to TCA cycle function. J. Biol. Chem..

[17] Oemer G., Lackner K., Muigg K., Krumschnabel G., Watschinger K., Sailer S. (2018). Molecular structural diversity of mitochondrial cardiolipins. Proc. Natl. Acad. Sci. U. S. A..

[18] Kiebish M.A., Han X., Cheng H., Chuang J.H., Seyfried T.N. (2008). Cardiolipin and electron transport chain abnormalities in mouse brain tumor mitochondria: lipidomic evidence supporting the Warburg theory of cancer. J. Lipid Res..

[19] Petrosillo G., Portincasa P., Grattagliano I., Casanova G., Matera M., Ruggiero F.M. (2007). Mitochondrial dysfunction in rat with nonalcoholic fatty liver Involvement of complex I, reactive oxygen species and cardiolipin. Biochim. Biophys. Acta.

[20] Ryan T., Bamm V.V., Stykel M.G., Coackley C.L., Humphries K.M., Jamieson-Williams R. (2018). Cardiolipin exposure on the outer mitochondrial membrane modulates alpha-synuclein. Nat. Commun..

[21] Sparagna G.C., Chicco A.J., Murphy R.C., Bristow M.R., Johnson C.A., Rees M.L. (2007). Loss of cardiac tetralinoleoyl cardiolipin in human and experimental heart failure. J. Lipid Res..

[22] Raja V., Joshi A.S., Li G., Maddipati K.R., Greenberg M.L. (2017). Loss of cardiolipin leads to perturbation of acetyl-CoA synthesis. J. Biol. Chem..

[23] Patel M.S., Nemeria N.S., Furey W., Jordan F. (2014). The pyruvate dehydrogenase complexes: structure-based function and regulation. J. Biol. Chem..

[24] Gudi R., Bowker-Kinley M.M., Kedishvili N.Y., Zhao Y., Popov K.M. (1995). Diversity of the pyruvate dehydrogenase kinase gene family in humans. J. Biol. Chem..

[25] Roche T.E., Baker J.C., Yan X., Hiromasa Y., Gong X., Peng T. (2001). Distinct regulatory properties of pyruvate dehydrogenase kinase and phosphatase isoforms. Prog. Nucleic Acid Res. Mol. Biol..

[26] Harris R.A., Bowker-Kinley M.M., Huang B., Wu P. (2002). Regulation of the activity of the pyruvate dehydrogenase complex. Adv. Enzyme Regul..

[27] Korotchkina L.G., Patel M.S. (1995). Mutagenesis studies of the phosphorylation sites of recombinant human pyruvate dehydrogenase. Site-specific regulation. J. Biol. Chem..

[28] Karpova T., Danchuk S., Kolobova E., Popov K.M. (2003). Characterization of the isozymes of pyruvate dehydrogenase phosphatase: implications for the regulation of pyruvate dehydrogenase activity. Biochim. Biophys. Acta.

[29] Turkan A., Gong X., Peng T., Roche T.E. (2002). Structural requirements within the lipoyl domain for the Ca2+-dependent binding and activation of pyruvate dehydrogenase phosphatase isoform 1 or its catalytic subunit. J. Biol. Chem..

[30] Huang B., Gudi R., Wu P., Harris R.A., Hamilton J., Popov K.M. (1998). Isoenzymes of pyruvate dehydrogenase phosphatase. J. Biol. Chem..

[31] Pettit F.H., Roche T.E., Reed L.J. (1972). Function of calcium ions in pyruvate dehydrogenase phosphatase activity. Biochem. Biophys. Res. Commun..

[32] Schlame M., Towbin J.A., Heerdt P.M., Jehle R., DiMauro S., Blanck T.J. (2002). Deficiency of tetralinoleoyl-cardiolipin in Barth syndrome. Ann. Neurol..

[33] Fatica E.M., DeLeonibus G.A., House A., Kodger J.V., Pearce R.W., Shah R.R. (2019). Barth syndrome: exploring cardiac metabolism with induced pluripotent stem cell-derived cardiomyocytes. Metabolites.

[34] Barth P.G., Valianpour F., Bowen V.M., Lam J., Duran M., Vaz F.M. (2004). X-linked cardioskeletal myopathy and neutropenia (Barth syndrome): an update. Am. J. Med. Genet. A..

[35] Greenwell A.A., Gopal K., Altamimi T.R., Saed C.T., Wang F.Q., Dakhili S.A.T. (2021). Barth syndrome-related cardiomyopathy is associated with a reduction in myocardial glucose oxidation. Am. J. Physiol. Heart Circ. Physiol..

[36] Planas-Iglesias J., Dwarakanath H., Mohammadyani D., Yanamala N., Kagan V.E., Klein-Seetharaman J. (2015). Cardiolipin interactions with proteins. Biophys. J..

[37] Lou W., Reynolds C.A., Li Y., Liu J., Huttemann M., Schlame M. (2018). Loss of tafazzin results in decreased myoblast differentiation in C2C12 cells: a myoblast model of Barth syndrome and cardiolipin deficiency. Biochim. Biophys. Acta Mol. Cell Biol. Lipids.

[38] Vassylyev D.G., Symersky J. (2007). Crystal structure of pyruvate dehydrogenase phosphatase 1 and its functional implications. J. Mol. Biol..

[39] Yan J., Lawson J.E., Reed L.J. (1996). Role of the regulatory subunit of bovine pyruvate dehydrogenase phosphatase. Proc. Natl. Acad. Sci. U. S. A..

[40] Yang D., Gong X., Yakhnin A., Roche T.E. (1998). Requirements for the adaptor protein role of dihydrolipoyl acetyltransferase in the up-regulated function of the pyruvate dehydrogenase kinase and pyruvate dehydrogenase phosphatase. J. Biol. Chem..

[41] Shan C., Kang H.B., Elf S., Xie J., Gu T.L., Aguiar M. (2014). Tyr-94 phosphorylation inhibits pyruvate dehydrogenase phosphatase 1 and promotes tumor growth. J. Biol. Chem..

[42] Lawson J.E., Niu X.D., Browning K.S., Trong H.L., Yan J., Reed L.J. (1993). Molecular cloning and expression of the catalytic subunit of bovine pyruvate dehydrogenase phosphatase and sequence similarity with protein phosphatase 2C. Biochemistry.

[43] Ghosh S., Zulkifli M., Joshi A., Venkatesan M., Cristel A., Vishnu N. (2022). MCU-complex-mediated mitochondrial calcium signaling is impaired in Barth syndrome. Hum. Mol. Genet..

[44] McKenzie M., Lim S.C., Duchen M.R. (2017). Simultaneous measurement of mitochondrial calcium and mitochondrial membrane potential in live cells by fluorescent microscopy. J. Vis. Exp..

[45] Pilotto F., Schmitz A., Maharjan N., Diab R., Odriozola A., Tripathi P. (2022). PolyGA targets the ER stress-adaptive response by impairing GRP75 function at the MAM in C9ORF72-ALS/FTD. Acta Neuropathologica.

[46] Treiman M., Caspersen C., Christensen S.B. (1998). A tool coming of age: thapsigargin as an inhibitor of sarco-endoplasmic reticulum Ca(2+)-ATPases. Trends Pharmacol. Sci..

[47] Ji J., Damschroder D., Bessert D., Lazcano P., Wessells R., Reynolds C.A. (2022). NAD supplementation improves mitochondrial performance of cardiolipin mutants. Biochim. Biophys. Acta Mol. Cell Biol. Lipids.

[48] Jeong K.-Y., Sim J.-J., Park M.H., Kim H.M. (2021). Remodeling of cancer-specific metabolism under hypoxia with lactate calcium Salt in human colorectal cancer cells. Cancers.

[49] Gherardi G., Monticelli H., Rizzuto R., Mammucari C. (2020). The mitochondrial Ca(2+) uptake and the fine-tuning of aerobic metabolism. Front. Physiol..

[50] Dowler S., Kular G., Alessi D.R. (2002). Protein lipid overlay assay. Sci. STKE.

